# The effect of *Zataria multiflora* Boiss hydroalcoholic extract and fractions in pentylenetetrazole-induced kindling in mice

**Published:** 2016

**Authors:** Ali Shamsizadeh, Farangis Fatehi, Fatemeh Arab Baniasad, Fatemeh Ayoobi, Mohammad Ebrahim Rezvani, Ali Roohbakhsh

**Affiliations:** 1 *Physiology-Pharmacology Research Center, Rafsanjan University of Medical Sciences, Rafsanjan, Iran*; 2 *Department of Physiology, School of Medicine, Shahid Sadoughi University of Medical Sciences, Yazd, Iran *; 3 *Pharmaceutical Research Center, School of Pharmacy, Mashhad University of Medical Sciences, Mashhad, Iran*

**Keywords:** *Seizure*, *Zataria multiflora Boiss*, *Plant extracts*, *Pentylenetetrazole*

## Abstract

**Objective:**

At present, there are many antiepileptic drugs with a wide range of side effects on the human body. It was assumed that *Zataria multiflora Boiss* (*Z. multiflora*) with sedative, anti-spasmodic and anti-inflammatory activity may be effective in the treatment of epilepsy. The aim of the present study was to elucidate the effect of *Z. multiflora* hydroalcoholic extract and its fraction extracts on pentylenetetrazole (PTZ)-induced chemical kindling.

**Materials and Methods:**

In this experimental study, eight separate groups of male albino mice were used. All groups received 11 separate intraperitoneal injections of PTZ (35 mg/kg) with two-day intervals. 30 min before the injection of PTZ, mice received vehicle, *Z. multiflora* hydroalcoholic extract (300 and 600 mg/kg), n-hexane, acetone, methanol fraction extracts (150 mg/kg), or diazepam (10 mg/kg).

**Results:**

The kindled mice that were pretreated with vehicle showed a gradual increase in their seizure scores up to the end of the study. The hydroalcoholic extract of *Z. multiflora* (300 and 600 mg/kg) reduced seizure scores significantly. However, n-hexane, acetone and methanol extracts did not affect seizure scores significantly.

**Conclusion:**

The present findings demonstrate that the hydroalcoholic extract of *Z. multiflora* did reduce the severity of seizure attacks in PTZ-induced chemical kindling in mice.

## Introduction

Epilepsy is a common neurological disorder recognized by unpredictable and episodic repeated seizures, which are induced by abnormal discharges of cerebral neurons. Different types of seizures are identified based on their clinical features. Seizures are mostly divided into two main categories: partial and generalized (Italiano et al., 2014 ▶). In partial seizures, attacks have a localized onset in the brain, while in generalized seizures they have a distributed onset. This disorder has physical, psychological and socioeconomic side effects that further affect patients’ quality of life. According to the previous reports, 0.84 to 1.54 percent of people worldwide have epilepsy (Helmers et al., 2015 ▶ and Bell et al., 2014 ▶). Prevention of recurrent seizures is the main goal in the treatment of epilepsy. Because of the refractory epilepsies, adverse effects of the existing antiepileptic medications and inability of the current medications in correcting abnormalities that induce epilepsy, finding new medications for epilepsy is of great importance (Sucher and Carles, 2015 ▶). In recent years, interest in finding herbal-based medications for the treatment of various diseases, including epilepsy, has been growing (Sucher and Carles, 2015 ▶). Previous evidence showed that some herbal medications are useful in the treatment of epilepsy (Zhu et al., 2014 ▶).

*Zataria multiflora* Boiss (*Z. multiflora*) is a thyme-like plant. *Z. multiflora* grows only in southern and central Iran, Afghanistan and Pakistan (Sajed et al., 2013 ▶). The plant has phytochemicals similar to Thymus vulgaris, a well-known and widely used medicinal plant. It has low toxicity in in vivo studies, and has been largely used as a condiment in the food industry (Sajed et al., 2013 ▶). This plant has several medicinal uses including treatment of fever, premature labor pain, bone and joint pain, headache, migraine, common cold, bloating, nausea, and diarrhea (Naghibi et al., 2005 ▶). *Z. multiflora* extract has a prominent effect in animal models of lung diseases such as chronic obstructive pulmonary disease (Boskabady and Gholami Mahtaj, 2014 ▶). 

Animal models of epilepsy have been used extensively for finding better antiepileptic drugs. Chemical kindling is a model of epileptic seizures. In this model, seizure is induced by repeated administration of an initially sub-convulsive chemical such as pentylenetetrazole (PTZ) (Hansen et al., 2004 ▶). These administrations decrease seizure threshold and culminate in a generalized seizure (Hansen et al., 2004 ▶). Kindling is a valuable experimental model for complex partial epilepsy in patients (Kupferberg, 2001 ▶), and is considered as a drug resistant model of epilepsy (Loscher et al., 1993 ▶). *Z. multiflora* extract is able to inhibit calcium channels (Gharib Naseri, 2003 ▶). This inhibition leads to anticonvulsant effects in vivo (Damasceno et al., 2012 ▶). Considering this finding and previous evidence regarding the anticonvulsive (Mandegary et al., 2013 ▶) and sedative effects (Sharif Rohani et al., 2008 ▶) of *Z. multiflora*, we aimed to assess the effect of the extract of this plant on PTZ-induced chemical kindling.

## Materials and Methods

**Plant material and preparation of extracts**

*Z. multiflora* aerial parts were collected during March 2011 from Isfahan Botany Herbarium, and were identified by Dr. Valiollah Mozaffarian at Botany Research Division, Research Institute of Forests and Rangelands, Tehran, Iran. A voucher specimen has been kept in Isfahan Botany Herbarium (voucher specimen no. F-1-8-4-21). For preparation of the hydroalcoholic extract, 250 g of dried and powdered *Z. multiflora* was macerated with 80% ethanol (80% ethanol – 20% water). To obtain the non-polar, semi-polar and polar extract fractions, powdered sample of *Z. multiflora* was extracted with n-hexane three times during 72 h with constant stirring. The remaining solid material was then extracted with acetone and then with methanol with the same procedure at room temperature (Molina-Salinas et al., 2006 ▶). The solvents were eliminated from the extracts by evaporation under vacuum in a rotary evaporator. The crude extract and its fractions were stored at -20 ^o^C before experiments. 

**Animals**

Male albino mice weighing 25–35 g were used in this study. Animals were housed six per cage, in a room with a 12:12 h light/dark cycle (lights on at 07:00 h) and controlled temperature (23 ± 2 ^o^C). Animals had free access to food and water. The method of this study was approved by the Ethics and Animal Care Committee of Rafsanjan University of Medical Sciences.

**Induction of kindling and experimental design**

For induction of kindling, PTZ (35 mg/kg) was injected intraperitoneally every other day for 20 days (Ben et al., 2014 ▶). The vehicle (10 ml/kg), hydroalcoholic extract (300 and 600 mg/kg), fraction extracts (150 mg/kg), and diazepam (as control, 10 mg/kg), all were administered intraperitoneally 30 min before PTZ injections. The doses for hydroalcoholic extract and diazepam were selected according to studies of Arzi et al., 2003 and Crestani et al., 2000 respectively. In injection days, each mouse was placed in a Plexiglas box and its behavior was observed for 30 min to record the incidence of convulsions. The intensity of the seizure response was scored on the following scale: 0=no response; 1=vibrissae twitching, mouth and facial jerks; 2=myoclonic body jerks or head nodding; 3=forelimb clonus; 4=rearing, falling down, forelimb tonus, and hindlimb clonus; and 5=tonic extension of hindlimb, status epilepticus (Jain et al., 2011 ▶). The highest response was recorded for each animal for each day.

**Statistical analysis**

The means of seizure scores were recorded in experimental groups. Repeated measurement analysis of variance (RMA) followed by Dunnett post hoc test was used for comparing the seizure scores in different times in each group. For comparison scores between different groups, one-way ANOVA followed by Dunnett post hoc test was used. Data are expressed as means ± S.E.M of six animals per group. p<0.05 was considered statistically significant.

## Results

**The effect of** ***Z. multiflora*** **hydroalcoholic extract on PTZ-induced kindling**

The results showed that repeated administration of PTZ for 20 days (control group) gradually decreased seizure threshold, which was manifested as increased seizure scores (p<0.01).

**Figure 1:**
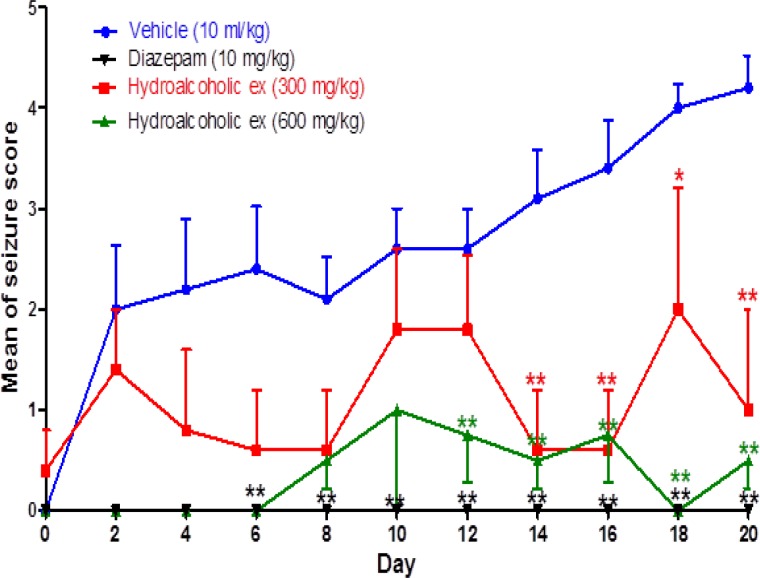
The effect of different doses of Zataria multiflora Boiss hydroalcoholic extract and diazepam on the mean of seizure score in PTZ kindled rats. Values are mean ± S.E.M. *: p<0.05 and **: p<0.01 when compared with the respective day in the vehicle-injected control group

In animals that were pretreated with hydroalcoholic extract of *Z. multiflora* (300 and 600 mg/kg), the seizure scores were not statistically different from day 0 to 20. Moreover, comparing the seizure scores between these animals with the control group (day to day comparison) showed significant differences in seizure scores in days 14-20 for 300 mg/kg extract and days 6-20 for 600 mg/kg extract (p<0.01). These data indicated that hydroalcoholic extract of *Z. multiflora* inhibited the development of PTZ-induced chemical kindling (figure 1). Diazepam as control drug at the dose of 10 mg/kg had a significant effect in controlling seizure scores (p< 0.01, Figure 1).

**The effect of** ***Z. multiflora*** **fractionated extracts on PTZ-induced kindling**

**Figure 2:**
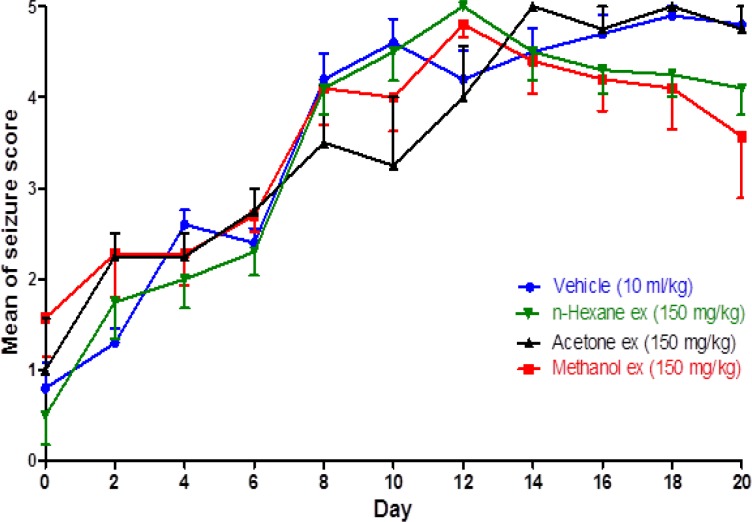
The effect of different fractions of Zataria multiflora Boiss on the mean of seizure scores in PTZ kindled rats. Values are mean ± S.E.M

The effects of n-hexane, acetone and methanol extracts on PTZ-induced kindling are presented in Figure 2. In these animals repeated administration of PTZ for 20 days gradually increased seizure scores (p<0.001). 

Moreover, comparing the seizure scores between these animals with the control group (day to day comparison), showed no significant difference in seizure scores in any of the 20 experimental days (p>0.05). These data indicated that in contrast to the results of the total plant extract (hydroalcoholic), n-hexane, acetone and methanol extracts did not inhibit the development of PTZ-induced chemical kindling (Figure 2).

## Discussion

The main finding of the present study is that hydroalcoholic extract of *Z. multiflora* had an anticonvulsant effect, while unexpectedly its n-hexane, acetone and methanol fraction extracts did not show any significant effect in PTZ kindled mice. This finding suggests that a mixture of different chemical compounds in the hydroalcoholic extract was able to reduce seizure score, whereas separation of these compounds by fractionation reduced the anticonvulsant effect of the plant extract. In agreement with the present finding, the anticonvulsant effect of total plant extract (methanolic) and essential oil of *Z. multiflora* against PTZ- and maximal electroshock- induced convulsions was reported in a recent study (Mandegary et al., 2013 ▶). The main difference between the present study and the previous one is the animal model that was employed. Furthermore, neither of the previous studies evaluated the effect of *Z. multiflora* fractions on experimental models of seizure. In the present study a low and non-convulsant dose of PTZ (35 mg/kg) gradually induced seizure attacks resembling complex partial epilepsy (Kupferberg, 2001 ▶), while administration of a high dose of PTZ (100 mg/kg) in the other study induced convulsions similar to absence and myoclonic seizures in humans (Loscher and Schmidt, 1988 ▶). The protective effects of *Z. multiflora* against maximal electroshock in Mandegary and coworkers’ study, may imply that the total extract is also able to reduce generalized tonic-clonic seizures in clinical practice (Loscher et al., 1991 ▶). Furthermore, it has been reported that *Z. multiflora* hydroalcoholic extract is able to reduce nicotine-induced convulsions (Arzi et al., 2003 ▶). These findings suggest that *Z. multiflora* is a plant with possible broad anticonvulsant effects that needs further basic and clinical studies. However, in the present study, fractionation of the extract, as discussed previously, did not produce such anticonvulsant effects. This implies that the total extract should be considered for future studies. The main active constituents of *Z. multiflora* are thymol, carvacrol, p-cymene, γ-terpinene, linalool apigenin, luteolin, 6-hydroxyluteolin, and β-sitosterol (Sajed et al., 2012 ▶). Some of these natural chemicals have significant protective effects in animal models of seizure. For example, in a recent study it was demonstrated that thymol had anticonvulsant effects in PTZ-induced chemical kindling (Sancheti et al., 2014 ▶). Another study revealed that carvacrol reduced latency to seizure both in PTZ- and electroshock-induced convulsions (Quintans-Júnior et al. 2013 ▶). Such anticonvulsant effects have also been reported for apigenin (Han et al., 2012 ▶). Hence, we suggest that these compounds possibly participated, at least in part, in the anticonvulsant effect of *Z. multiflora* ethanolic extract. 

Similar to *Z. multiflora* hydroalcoholic extract, there are other plants with good anticonvulsant effects such as Valeriana officinalis (Rezvani et al., 2010 ▶) and *Rosa damascena* (Homayoun et al., 2015 ▶). Because of limited geographical distribution of *Z. multiflora*, the pharmacological effects of the extract have not been evaluated in detail. Hence, it is hard to suggest the molecular mechanisms behind the anticonvulsant effect of *Z. multiflora* hydroalcoholic extract. One explanation is blockade of calcium channels; previous studies showed that inhibition of L-type calcium channels can reduce seizure activity in vivo (Damasceno et al., 2012 ▶). Moreover, hydroalcoholic extract of *Z. multiflora* through inhibition of calcium channels had a spasmolytic effect on rat ileum (Naseri, 2003 ▶). Thus, it is a possibility that *Z. multiflora* extract through inhibition of these channels reduced convulsions. It has been reported that following a single injection of PTZ or PTZ-induced kindling, the brain antioxidant system changed significantly (Erakovic et al., 2003 ▶). Accordingly, recent studies are considering oxidative stress as an important factor in the pathophysiology of epilepsy (Martinc et al., 2014 ▶). Therefore, it is a possibility that *Z. multiflora*, through inhibition of oxidative stress (Boskabady and Gholami Mahtaj, 2015 ▶), decreased the seizure score in the present study. It may also be suggested that a combination of antioxidants in different fractions was necessary to induce such antiepileptic effects. On the other hand, *Z. multiflora* essential oil had beneficial effects in a mouse model of Alzheimer’s disease (Majlessi et al., 2012 ▶). The anti-cholinesterase effects of *Z. multiflora* essential oil have also been reported separately (Sharififar et al., 2012 ▶). Tacrine, as an important drug in the treatment of Alzheimer’s disease, is able to reduce the deteriorative effect of PTZ on memory during kindling (Getova and Dimitrova, 2000 ▶), therefore it may be suggested that *Z. multiflora* may also be useful in the treatment of memory failure in epileptic patients (Piazzini et al., 2001 ▶). In the present study, we did not evaluate the effect of the extract and its fractions on motor coordination and locomotor activity of the animals as potential confounding factors. It is a possibility that *Z. multiflora* through inhibition of locomotor activity and/or change in muscular tone changed the seizure scores. Although this hypothesis was rejected by Mandegari et al. (2013) ▶, who showed that administration of the methanolic extract of *Z. multiflora* up to 2 g/kg did not change the rotarod performance of the mice. In conclusion, *Z. multiflora* had an anticonvulsant effect in PTZ induced kindling in mice. However, fractionation of the total extract did not induce such effects.
